# Systematic Characterization of Prognostic Values of Peroxiredoxin Family in Gastric Cancer

**DOI:** 10.1155/2020/3948183

**Published:** 2020-01-09

**Authors:** Rui Xu, Jiadong Pan, Jie Mei, Qinglin Zhang

**Affiliations:** ^1^School of Basic Medicine, Nanjing Medical University, Nanjing 211166, China; ^2^Department of Gastroenterology, Wuxi People's Hospital Affiliated to Nanjing Medical University, Wuxi 214023, China; ^3^Department of Oncology, Wuxi People's Hospital Affiliated to Nanjing Medical University, Wuxi 214023, China

## Abstract

The peroxiredoxin (PRDX) gene family has been reported to participate in regulating occurrence and development of cancerous diseases, but its exact prognostic values in gastric cancer (GC) remain largely elusive. In the current research, we evaluated the prognostic value in predicting overall survival (OS) of each individual PRDX mRNA expression based on patients' cohorts from the Kaplan–Meier (KM) plotter database, which contains clinical information and gene expression data obtained from a total of 876 GC patients. Our results revealed that mRNA expressions of PRDX1, PRDX2, PRDX3, and PRDX4 were significantly associated with worse OS in GC patients, whereas PRDX5 and PRDX6 mRNA expressions were not associated with OS in GC patients. In addition, the prognostic values of PRDXs in the different clinicopathological features according to clinical stages, Lauren classifications, HER2 expression status, differentiation degree, and treatment strategies of GC patients were further evaluated in the KM plotter database. As a result, more potential beneficiaries who may benefit from prognostic assessment using PRDX mRNA expressions were identified. Our results elucidated the exact values of PRDXs in assessing GC prognosis and might provide primary evidence for further study on the mechanism of PRDXs participating in occurrence and development of GC.

## 1. Introduction

Gastric cancer (GC) is one of the most common digestive malignancies worldwide; although its incidence has declined over the past century, it is still a major risk factor threatening human health. According to statistics, about 990,000 people are diagnosed with GC every year in the world, whose morbidity is ranked fourth in all malignant tumors [1]. According to the latest prediction of the American Cancer Society (ACS), there will be 27,510 new cases of GC and more than 11,000 deaths in the United States in 2019 [2]. Similar to other malignancies, the occurrence of GC is also a multifactor process, such as age, smoking, and *Helicobacter pylori* infection, leading to abnormal activation of oncogene and inactivation of tumor suppressor at last [3, 4]. At present, the research on the carcinogenesis of GC is still lacking. In order to innovate the clinical therapies for GC and improve the prognosis of GC patients, it is necessary to further explore the potential molecular mechanisms in the carcinogenesis and progression of GC, which may help identify possible therapeutic targets and prognostic biomarkers.

Peroxiredoxins (PRDXs) are antioxidant enzymes which can neutralise a wide range of reactive oxygen species (ROS), such as hydrogen peroxide (H_2_O_2_) and alkyl hydroperoxides. The PRDX family contains six isoforms: PRDX1, PRDX2, PRDX3, PRDX4, PRDX5, and PRDX6. As mediators in regulating ROS levels, PRDXs are localized to the cytoplasm and reduce H_2_O_2_ and alkyl hydroperoxides to water and alcohol with the use of reducing equivalents derived from thiol-containing donor molecules [5]. An increasing number of studies have discovered several primary mechanisms of PRDXs participating in cancerous diseases [6–8]. Several studies have also identified the prognostic roles of PRDXs in multiple cancers, including lung cancer [9], ovarian cancer [10], and breast cancer [11]. However, the exact prognostic values of PRDXs in GC have not been explored up to date.

The Kaplan–Meier (KM) plotter (http://kmplot.com/analysis/) is a user-friendly online website and is capable of evaluating the impacts of total 54k genes on patient's survival in 21 cancer types [12]. The primary purpose of the tool is a meta-analysis-based discovery and validation of survival biomarkers. In the current study, we applied the KM plotter database to evaluate the prognostic impacts of each PRDX mRNA expression in patients with GC. Finally, we identified PRDX1, PRDX2, PRDX3, and PRDX4 as promising prognostic candidates for monitoring GC patients and explored the potential beneficiaries by subgroup survival analysis.

## 2. Materials and Methods

### 2.1. Survival Analysis Based on KM Plotter

In this study, we employed online KM plotter (http://kmplot.com/analysis) database to evaluate the prognostic values of PRDX mRNA expression for overall survival (OS) in GC patients. This database was established by collecting gene expression data and survival information from Gene Expression Omnibus (GEO), European Genome-phenome Archive (EGA), and the Cancer Genome Atlas (TCGA). The database totally contained clinical and gene expression information of 876 GC patients, and the clinical information comprised clinical stages, Lauren classifications, HER2 expression status, differentiation degree, and treatment strategies. In our study, the prognostic values of each PRDX mRNA expression in GC were assessed by the KM plotter database. In addition, we evaluated the correlations between OS of GC patients and PRDX expression according to different clinicopathological features. What needs to further explain was that the expression cutoff points of the PRDX mRNA were determined according to the median level of the gene from the selected GC specimens. GC samples were split into “low expression group” and “high expression group” depending on the comparisons between expression levels with established cutoffs.

### 2.2. Statistical Analysis

All statistical analyses were performed on the KM plotter database online. For all survival analyses, patients' cohorts were compared with KM survival plots. Hazard ratio (HR), 95% confidence interval (95% CI), and logrank *P* value were calculated and displayed online. Differences were deemed to be statistically significant when *P* values were less than 0.05.

## 3. Results

### 3.1. Prognostic Values of PRDX mRNA Expression in all GC Patients

**Figure 1:**
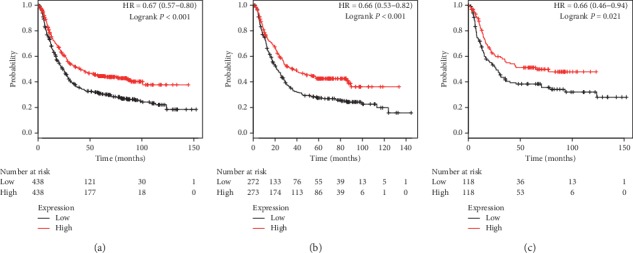
The prognostic value of PRDX1 expression in KM plotter. The valid Affymetrix ID is 208680_at (PRDX1). Survival curves are plotted for (a) all patients (*n* = 876), HR = 0.67 (95% CI: 0.57–0.80); (b) male patients (*n* = 545), HR = 0.66 (95% CI: 0.53–0.82); and (c) female patients (*n* = 236), HR = 0.66 (95% CI: 0.46–0.94).

The prognostic values of PRDX1 mRNA expression were firstly evaluated in the database. OS curves were plotted for all GC patients. Low expression of PRDX1 mRNA level exhibited a notable association with worse OS in total 876 GC patients (*P* < 0.001, HR = 0.57, 95% CI: 0.48–0.68, Figure 1(a)). The subtype analysis of different genders showed that decreased PRDX1 mRNA expression was associated with poor OS in both male (*P* < 0.001, HR = 0.66, 95% CI: 0.53–0.82, Figure 1(b)) and female (*P*=0.02, HR = 0.66, 95% CI: 0.46–0.94, Figure 1(c)) patients.

**Figure 2:**
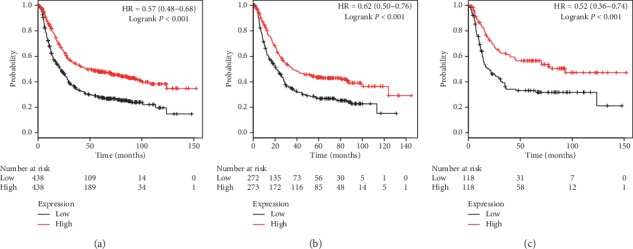
The prognostic value of PRDX2 expression in KM plotter. The valid Affymetrix ID is 39729_at (PRDX2). Survival curves are plotted for (a) all patients (*n* = 876), HR = 0.57 (95% CI: 0.48–0.68); (b) male patients (*n* = 545), HR = 0.62 (95% CI: 0.50–0.76); and (c) female patients (*n* = 236), HR = 0.52 (95% CI: 0.36–0.74).

The next evaluation for prognostic values of PRDX2 mRNA expression was performed in the database. Decreased PRDX2 mRNA expression was significantly associated with unfavourable OS in all GC patients (*P* < 0.001, HR = 0.57, 95% CI: 0.48–0.68, Figure 2(a)), male patients (*P* < 0.001, HR = 0.62, 95% CI: 0.50–0.76, Figure 2(b)), and female patients (*P* < 0.001, HR = 0.52, 95% CI: 0.36–0.74, Figure 2(c)). As the results showed, female GC patients with high PRDX2 mRNA expression exhibited a 48% reduction in risk of death, which suggested prognostic evaluation using PRDX2 mRNA expression had additional value in female patients compared with male patients.

**Figure 3:**
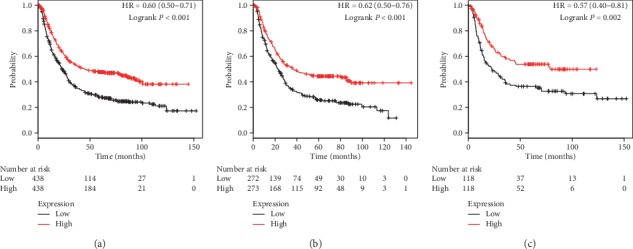
The prognostic value of PRDX3 expression in KM plotter. The valid Affymetrix ID is 201619_at (PRDX3). Survival curves are plotted for (a) all patients (*n* = 876), HR = 0.60 (95% CI: 0.50–0.71); (b) male patients (*n* = 545), HR = 0.62 (95% CI: 0.50–0.76); and (c) female patients (*n* = 236), HR = 0.57 (95% CI: 0.40–0.81).

Figure 3 shows the prognostic values of PRDX3 in the database. Low PRDX3 mRNA expression was significantly associated with worse OS in all GC patients (*P* < 0.001, HR = 0.60, 95% CI: 0.50–0.71, Figure 3(a)), male patients (*P* < 0.001, HR = 0.62, 95% CI: 0.50–0.76, Figure 3(b)), and female patients (*P*=0.002, HR = 0.57, 95% CI: 0.40–0.81, Figure 3(c)).

**Figure 4:**
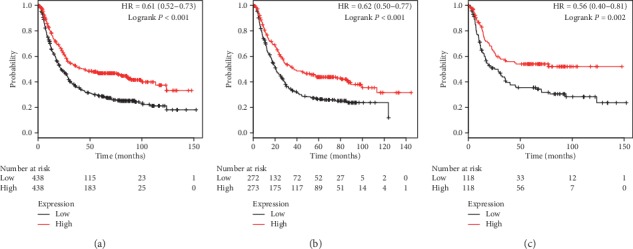
The prognostic value of PRDX4 expression in KM plotter. The valid Affymetrix ID is 201923_at (PRDX4). Survival curves are plotted for (a) all patients (*n* = 876), HR = 0.61 (95% CI: 0.52–0.73); (b) male patients (*n* = 545), HR = 0.62 (95% CI: 0.50–0.77); and (c) female patients (*n* = 236), HR = 0.56 (95% CI: 0.40–0.81).

Figure 4 illustrates prognostic impacts of PRDX4 mRNA expression in the database. Similar to the first three family members, low PRDX4 mRNA levels were remarkably correlated with poor OS in all GC patients (*P* < 0.001, HR = 0.61, 95% CI: 0.52–0.73, Figure 4(a)), male patients (*P* < 0.001, HR = 0.62, 95% CI: 0.50–0.77, Figure 4(b)), and female patients (*P*=0.002, HR = 0.56, 95% CI: 0.40–0.81, Figure 4(c)).

**Figure 5:**
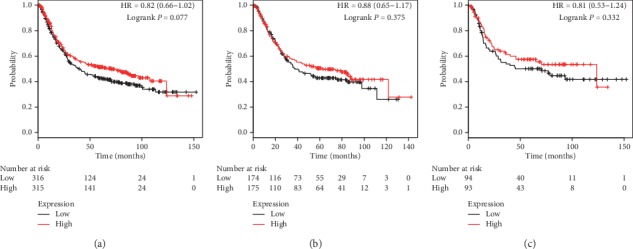
The prognostic value of PRDX5 expression in KM plotter. The valid Affymetrix ID is 1560587_s_at (PRDX5). Survival curves are plotted for (a) all patients (*n* = 631), HR = 0.82 (95% CI: 0.66–1.02); (b) male patients (*n* = 349), HR = 0.88 (95% CI: 0.65–1.17); and (c) female patients (*n* = 187), HR = 0.81 (95% CI: 0.53–1.24).

**Figure 6:**
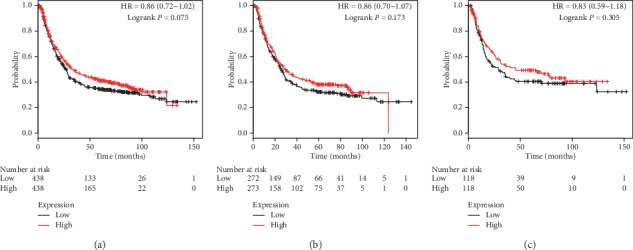
The prognostic value of PRDX6 expression in KM plotter. The valid Affymetrix ID is 200845_s_at (PRDX6). Survival curves are plotted for (a) all patients (*n* = 876), HR = 0.86 (95% CI: 0.72–1.02); (b) male patients (*n* = 545), HR = 0.86 (95% CI: 0.70–1.07); and (c) female patients (*n* = 236), HR = 0.83 (95% CI: 0.59–1.18).

Figures 5 and 6 present prognostic association of PRDX5 and PRDX6 mRNA expression in the database, respectively. Different from the above family members, mRNA expression of both PRDX5 and PRDX6 had no obvious association with clinical outcomes. Detailed parameters are as follows: prognostic values of PRDX5 in all patients (*P*=0.077, HR = 0.82, 95% CI: 0.66–1.02, Figure 5(a)), male patients (*P*=0.375, HR = 0.88, 95% CI: 0.65–1.17, Figure 5(b)), and female patients (*P*=0.332, HR = 0.81, 95% CI: 0.53–1.24, Figure 5(c)), and prognostic values of PRDX6 in all patients (*P*=0.075, HR = 0.86, 95% CI: 0.72–1.02, Figure 6(a)), male patients (*P*=0.173, HR = 0.86, 95% CI: 0.70–1.07, Figure 6(b)), and female patients (*P*=0.305, HR = 0.83, 95% CI: 0.59–1.18, Figure 6(c)).

### 3.2. Subgroup Analysis of PRDXs' Prognostic Values in GC Patients according to Clinicopathological Characteristics

In addition to our assessments of the prognostic values of PRDX mRNA expressions in general GC patients, we further performed subtype analysis to assess the associations with different clinicopathological characteristics to identify more potential beneficiaries who may benefit from prognostic assessment using PRDX mRNA expressions, according to clinical stages, Lauren classifications, HER2 expression status, differentiation degree, and treatment strategies.

As presented in Table 1, we found that low expressions of PRDX1, PRDX2, PRDX3, and PRDX4 were significantly correlated with unfavourable OS in stage III GC patients. Besides, decreased expressions of PRDX1 and PRDX4 had a notable relationship to poor OS in stage IV GC patients as well. These results suggested GC patients with advanced stages (III and IV) tend to benefit from prognostic assessment using PRDX mRNA expressions.

In Table 2, we further investigated the association between PRDX expression and Lauren classifications in GC patients. The results showed that low expressions of PRDX1, PRDX2, PRDX3, and PRDX6 were remarkably correlated with poor OS in patients with intestinal GC. Besides, decreased PRDX1 and PRDX3 showed correlations with worse OS in patients with diffuse GC as well. The reason why no PRDX expressions associated with OS in mixed GC may be due to the limited number of mixed patients (only 32 patients with mixed GC).

Next, Table 3 reveals correlations of PRDX expressions with OS according to different HER2 status in GC patients. Both positive HER2 status and negative HER2 status were associated with poor OS in PRDX1, PRDX2, and PRDX4 mRNA expressions in GC patients. However, decreased PRDX3 only showed correlations with worse OS in patients with negative HER2 status GC. Moreover, PRDX5 and PRDX6 had no correlation with OS in both positive and negative HER2 status in GC patients.

Table 4 demonstrates correlations of PRDX expression with OS according to various differentiation degrees in GC patients. We found that GC patients with low expressions of PRDX2 with moderate differentiation exhibited worse OS. However, all other PRDX expressions showed no significance in OS in GC patients with different differentiation degrees. Although several positive findings were observed, we believe that the evidence provided by this subtype analysis is not strong due to the most cases in KM plotter missing the information of differentiation degrees.

Last but not least, the results in Table 5 exhibited the correlation of PRDX mRNA expressions with OS based on different treatment strategies in GC patients. The results revealed that expressions of PRDX2 and PRDX3 showed worse OS in GC patients treated with surgery alone. Subsequent analysis indicated that low expression of PRDX3 was also associated with poor OS in GC patients receiving 5-FU-based adjuvant chemotherapy regimens. Regretfully, all other PRDX expressions exhibited no significance in OS in GC patients receiving different treatment strategies.

## 4. Discussion

PRDXs are one of the most significant antioxidant enzyme systems, which include superoxide dismutase (SOD), catalase (CAT), and glutathione peroxidase (GPX). PRDXs tend to be remarkably overexpressed when cells are under oxidative stress conditions and mainly participate in the defense against oxidative environment [13, 14]. In the carcinogenesis of cancerous diseases, interestingly, several studies showed the double effects of PRDXs in the carcinogenesis [15]. Namely, overexpression of PRDXs may either inhibit cancer development or promote cancer growth, depending on the specific PRDX family member and the types of cancers.

Although recent literature studies reveal the prognostic roles of PRDXs in several cancers, the prognostic values in GC have not yet been evaluated. In the current study, we explored the prognostic roles of PRDX mRNA expression in GC patients based on the data gathered from the KM plotter. Our results revealed that decreased expression of PRDX1, PRDX2, PRDX3, and PRDX4 family members was significantly correlated with unfavourable survival in total patients suffering from GC. However, no additional value was found in PRDX5 and PRDX6 in predicting the prognosis of all GC patients.

PRDX1 is an antioxidant enzyme that can act as a promotor in inflammatory response [16]. Recent studies have shown that PRDX1 functioning as a potential oncogene was observed in numerous cancers, including ovarian cancer [17], head and neck squamous cell carcinoma [18], and breast cancer [19]. An observational research based on 189 GC cases suggested that overexpression of PRDX1 predicted poor OS in GC, and further research on mechanism revealed PRDX1 promotes GC cell invasion and metastasis through epithelial-mesenchymal transition- (EMT-) dependent mechanisms [20]. This seems to be inconsistent with our findings that low PRDX1 expression was associated with poor OS, but double effects of PRDXs on cancer cells have been identified previously [15].

PRDX2 is crucial to multiple cell processes, including cell migration, proliferation, differentiation, and carcinogenesis [21, 22]. PRDX2 is an important member of the ROS scavenging system, and deletion of PRDX2 promotes age-related ovarian failure via the ROS-mediated JNK pathway *in vivo* [23]. Several studies have observed the oncogene role in numerous cancers [24, 25]. However, no exact functions of PRDX2 in GC are currently established. In our research, we revealed decreased PRDX2 mRNA expression was significantly associated with unfavourable OS in GC patients, suggesting the tumor suppressor role of PRDX2 in GC.

According to previous publications, high expression PRDX3 is associated with advanced malignant phenotype and worse prognosis in hepatocellular carcinoma [26], endometrial cancer [27], and medulloblastoma [28]. Besides, serum proteomics-based analysis identified autoantibodies against PRDX3 as potential diagnostic biomarkers in nasopharyngeal carcinoma [29]. In GC, our results found that decreased PRDX3 was correlated with worse OS in all GC patients, and subgroup analysis revealed PRDX3 only was associated with poor OS in patients with negative HER2 status GC.

PRDX4 is a PRDX family member which has an exact double effect on cancer cells according to the previous publication. Guo et al. revealed that deletion of PRDX4 enhanced the risk of diethylnitrosamine- (DEN-) induced hepatocellular carcinoma in mice and low expression PRDX4 was significantly associated with poor prognosis in hepatocellular carcinoma patients. However, decreased PRDX4 repressed cell proliferation and triggered cell death pathways in hepatocellular carcinoma cell lines [15], suggesting the potential role of PRDX4 as activators or inhibitors in hepatocellular carcinoma with different stages and phenotypes. In GC, PRDX4 had been reported to be decreased and served as a biomarker candidate to diagnose GC [30]. Based on the current research, we discovered low PRDX4 mRNA expression was significantly associated with worse OS in GC patients, suggesting the tumor suppressor role of PRDX4 in GC.

PRDX5 and PRDX6 are other significant members of PRDX family. Numerous studies have revealed the potential oncogenic roles in cancers. It is believed that PRDX5 overexpression enhanced tumorigenesis and predicted poor prognosis in GC [31]. However, our research showed that mRNA expression of both PRDX5 and PRDX6 had no obvious association with clinical outcomes. Interestingly, low PRDX6 mRNA expression showed a significant correlation with worse OS in stage III GC patients. The reason why there were different phenomena in prognostic of PRDX5 and PRDX6 in patients with GC might be due to randomness of patients' cohort.

## 5. Conclusion

In this study, we systemically evaluated the prognostic values of six PRDX members in patients with GC using the KM plotter database. As results, we discovered that low PRDX1–4 mRNA expressions were significantly associated with deteriorated OS in GC patients, whereas PRDX5 and PRDX6 mRNA expressions had no association with OS in GC patients. In summary, our findings gave new insights into the prognostic values of PRDX mRNA and provided primary evidences that PRDXs are involved in the mechanism of carcinogenesis of GC.

## Figures and Tables

**Table 1:** Association between PRDX expression and OS in GC patients at different clinical stages.

PRDXs	Clinical stages	Cases	Low	High	HR (95% CI)	*P* value
PRDX1	I	67	34	33	0.51 (0.17–1.52)	0.217
	II	140	70	70	0.80 (0.43–1.46)	0.462
	III	315	152	153	0.53 (0.40–0.71)	<0.001
	IV	148	74	74	0.57 (0.39–0.84)	0.004

PRDX2	I	67	34	33	1.15 (0.43–3.08)	0.787
	II	140	70	70	0.79 (0.43–1.44)	0.439
	III	315	152	153	0.53 (0.40–0.71)	<0.001
	IV	148	74	74	0.79 (0.54–1.15)	0.219

PRDX3	I	67	34	33	0.51 (0.17–1.52)	0.218
	II	140	70	70	0.79 (0.43–1.44)	0.749
	III	315	152	153	0.62 (0.46–0.82)	<0.001
	IV	148	74	74	0.86 (0.58–1.25)	0.425

PRDX4	I	67	34	33	0.75 (0.28–2.03)	0.571
	II	140	70	70	0.94 (0.51–1.72)	0.836
	III	315	152	153	0.67 (0.50–0.89)	0.005
	IV	148	74	74	0.66 (0.45–0.96)	0.031

PRDX5	I	62	31	31	0.93 (0.31–2.78)	0.897
	II	135	68	67	1.41 (0.74–2.68)	0.294
	III	197	98	99	1.03 (0.71–1.50)	0.866
	IV	140	70	70	0.92 (0.62–1.36)	0.660

PRDX6	I	67	34	33	0.63 (0.23–1.74)	0.367
	II	140	70	70	0.92 (0.51–1.67)	0.787
	III	315	152	153	0.72 (0.54–0.96)	0.025
	IV	148	74	74	0.90 (0.62–1.32)	0.607

**Table 2:** Association between PRDX expression and OS in GC patients with different Lauren classifications.

PRDXs	Lauren classification	Cases	Low	High	HR (95% CI)	*P* value
PRDX1	Intestinal	320	160	160	0.56 (0.41–0.78)	<0.001
	Diffuse	241	120	121	0.59 (0.42–0.84)	0.003
	Mixed	32	16	16	0.75 (0.27–2.06)	0.571

PRDX2	Intestinal	320	160	160	0.47 (0.34–0.65)	<0.001
	Diffuse	241	120	121	0.99 (0.70–1.39)	0.949
	Mixed	32	16	16	1.24 (0.44–3.52)	0.680

PRDX3	Intestinal	320	160	160	0.59 (0.43–0.81)	<0.001
	Diffuse	241	120	121	0.66 (0.47–0.93)	0.017
	Mixed	32	16	16	0.53 (0.19–1.50)	0.222

PRDX4	Intestinal	320	160	160	0.76 (0.55–1.04)	0.088
	Diffuse	241	120	121	0.72 (0.51–1.01)	0.054
	Mixed	32	16	16	0.51 (0.18–1.44)	0.197

PRDX5	Intestinal	269	134	135	0.92 (0.64–1.32)	0.640
	Diffuse	240	120	120	0.85 (0.61–1.20)	0.360
	Mixed	29	14	15	1.22 (0.41–3.65)	0.721

PRDX6	Intestinal	320	160	160	0.67 (0.49–0.92)	0.012
	Diffuse	241	120	121	0.86 (0.61–1.21)	0.384
	Mixed	32	16	16	1.51 (0.54–4.26)	0.429

**Table 3:** Association between PRDX expression and OS in GC patients with different HER2 expression status.

PRDXs	HER2 status	Cases	Low	High	HR (95% CI)	*P* value
PRDX1	Negative	532	266	266	0.62 (0.49–0.77)	<0.001
	Positive	344	172	172	0.75 (0.58–0.98)	0.031

PRDX2	Negative	532	266	266	0.66 (0.52–0.82)	<0.001
	Positive	344	172	172	0.56 (0.43–0.73)	<0.001

PRDX3	Negative	532	266	266	0.51 (0.40–0.64)	<0.001
	Positive	344	172	172	0.97 (0.75–1.25)	0.809

PRDX4	Negative	532	266	266	0.63 (0.51–0.79)	<0.001
	Positive	344	172	172	0.72 (0.56–0.94)	0.014

PRDX5	Negative	429	214	215	0.89 (0.68–1.16)	0.390
	Positive	202	101	101	0.77 (0.53–1.12)	0.171

PRDX6	Negative	532	266	266	0.81 (0.65–1.01)	0.061
	Positive	344	172	172	1.05 (0.81–1.35)	0.737

**Table 4:** Association between PRDX expression and OS in GC patients with different differentiation degree.

PRDXs	Differentiation degree	Cases	Low	High	HR (95% CI)	*P* value
PRDX1	Poor	165	82	83	1.40 (0.94–2.08)	0.098
	Moderate	67	34	33	0.85 (0.44–1.63)	0.629
	Good	32	16	16	1.07 (0.46–2.54)	0.870

PRDX2	Poor	165	82	83	0.87 (0.58–1.30)	0.494
	Moderate	67	34	33	0.32 (0.16–0.63)	<0.001
	Good	32	16	16	0.61 (0.26–1.46)	0.268

PRDX3	Poor	165	82	83	1.06 (0.71–1.57)	0.792
	Moderate	67	34	33	0.57 (0.30–1.10)	0.091
	Good	32	16	16	0.66 (0.28–1.57)	0.344

PRDX4	Poor	165	82	83	1.25 (0.84–1.86)	0.271
	Moderate	67	34	33	0.59 (0.30–1.14)	0.113
	Good	32	16	16	0.48 (0.20–1.17)	0.099

PRDX5	Poor	121	60	61	1.15 (0.71–1.86)	0.573
	Moderate	67	34	33	0.82 (0.43–1.57)	0.544
	Good	5	2	3	—	0.221

PRDX6	Poor	165	82	83	0.88 (0.59–1.30)	0.512
	Moderate	67	34	33	1.10 (0.57–2.11)	0.775
	Good	32	16	16	1.33 (0.56–3.13)	0.518

**Table 5:** Association between PRDX expression and OS in GC patients with different treatment strategies.

PRDXs	Treatment strategies	Cases	Low	High	HR (95% CI)	*P* value
PRDX1	Surgery alone	380	190	190	0.76 (0.57–1.01)	0.057
	5-FU-based adjuvant	153	76	77	1.21 (0.86–1.71)	0.268
	Other adjuvants	76	38	38	0.67 (0.27–1.64)	0.374

PRDX2	Surgery alone	380	190	190	0.72 (0.54–0.96)	0.025
	5-FU-based adjuvant	153	76	77	0.68 (0.48–0.97)	0.030
	Other adjuvants	76	38	38	0.61 (0.25–1.50)	0.276

PRDX3	Surgery alone	380	190	190	0.74 (0.55–0.99)	0.038
	5-FU-based adjuvant	153	76	77	1.20 (0.85–1.69)	0.296
	Other adjuvants	76	38	38	0.94 (0.39–2.26)	0.883

PRDX4	Surgery alone	380	190	190	0.85 (0.64–1.14)	0.275
	5-FU-based adjuvant	153	76	77	0.78 (0.56–1.11)	0.164
	Other adjuvants	76	38	38	1.01 (0.42–2.43)	0.990

PRDX5	Surgery alone	380	191	189	0.82 (0.62–1.10)	0.187
	5-FU-based adjuvant	34	17	17	0.94 (0.38–2.33)	0.893
	Other adjuvants	76	38	38	0.48 (0.19–1.20)	0.110

PRDX6	Surgery alone	380	190	190	1.04 (0.78–1.38)	0.795
	5-FU-based adjuvant	153	76	77	0.98 (0.70–1.38)	0.913
	Other adjuvants	76	38	38	1.16 (0.48–2.80)	0.738

## Data Availability

The data used to support the findings of this study are available from the KM plotter database (http://kmplot.com/analysis).
