# Corrigendum to “Systematic Characterization of Prognostic Values of Peroxiredoxin Family in Gastric Cancer”

**DOI:** 10.1155/bmri/9897568

**Published:** 2025-09-15

R. Xu, J. Pan, J. Mei, Q. Zhang, “Systematic Characterization of Prognostic Values of Peroxiredoxin Family in Gastric Cancer,” *BioMed Research International* 2020 (2020): 3948183, https://doi.org/10.1155/2020/3948183

In the article, there is an error in Table 1. The total number of patients for Stage III PRDX1, PRDX2, PRDX3, PRDX4, and PRDX6 should be 305, not 315. The correct Table 1 is shown as follows:

**Table 1:** Association between PRDX expression and OS in GC patients at different clinical stages.

**PRDXs**	**Clinical stages**	**Cases**	**Low**	**High**	**HR (95% CI)**	***P* value**
PRDX1	I	67	34	33	0.51 (0.17–1.52)	0.217
	II	140	70	70	0.80 (0.43–1.46)	0.462
	III	305	152	153	0.53 (0.40–0.71)	<0.001
	IV	148	74	74	0.57 (0.39–0.84)	0.004

PRDX2	I	67	34	33	1.15 (0.43–3.08)	0.787
	II	140	70	70	0.79 (0.43–1.44)	0.439
	III	305	152	153	0.53 (0.40–0.71)	<0.001
	IV	148	74	74	0.79 (0.54–1.15)	0.219

PRDX3	I	67	34	33	0.51 (0.17–1.52)	0.218
	II	140	70	70	0.79 (0.43–1.44)	0.749
	III	305	152	153	0.62 (0.46–0.82)	<0.001
	IV	148	74	74	0.86 (0.58–1.25)	0.425

PRDX4	I	67	34	33	0.75 (0.28–2.03)	0.571
	II	140	70	70	0.94 (0.51–1.72)	0.836
	III	305	152	153	0.67 (0.50–0.89)	0.005
	IV	148	74	74	0.66 (0.45–0.96)	0.031

PRDX5	I	62	31	31	0.93 (0.31–2.78)	0.897
	II	135	68	67	1.41 (0.74–2.68)	0.294
	III	197	98	99	1.03 (0.71–1.50)	0.866
	IV	140	70	70	0.92 (0.62–1.36)	0.660

PRDX6	I	67	34	33	0.63 (0.23–1.74)	0.367
	II	140	70	70	0.92 (0.51–1.67)	0.787
	III	305	152	153	0.72 (0.54–0.96)	0.025
	IV	148	74	74	0.90 (0.62–1.32)	0.607

We apologize for this error.

